# Design and evaluation of a custom contoured pneumatic insole for active plantar pressure offloading

**DOI:** 10.1177/20556683261488849

**Published:** 2026-09-11

**Authors:** Veysel Erel, Allison Palomino, Yi-Ting Tzen, Lawrance A. Lavery, Dane K. Wukich, Muthu B. J. Wijesundara

**Affiliations:** 1 698005 The University of Texas at Arlington Research Institute, Fort Worth, TX, USA; 2 Department of Applied Clinical Research, 12334 UT Southwestern Medical Center, Dallas, TX, USA; 3 Department of Orthopaedic Surgery, 14742 UT Health San Antonio, San Antonio, TX, USA; 4 Department of Prosthetics and Orthotics, 12334 University of Texas Southwestern Medical Center, Dallas, TX, USA

**Keywords:** lower extremity wounds, diabetic foot ulcers, shoe insole, active offloading, custom insole, plantar pressure

## Abstract

**Introduction:**

Diabetic foot ulcers (DFUs) are a major cause of morbidity, amputation, and mortality in people with diabetes. Excessive and repetitive plantar loading contributes significantly, and although therapeutic footwear is widely prescribed, its effectiveness varies. Custom insoles improve pressure redistribution but do not address repetitive loading. Emerging active offloading technologies offer targeted relief, yet fully custom-contoured active systems remain untested.

**Methods:**

This study compared a custom-contoured active insole with a non-custom flat active insole under static and dynamic conditions in an individual without DFU risk. Both designs used seven pneumatically actuated air cells and were tested at 1.5 and 2.0 psi. Pressures were measured using a high-resolution in-shoe sensor and quantified using maximum mean pressure (MMP) and peak pressure (PP).

**Results:**

The custom insole consistently produced lower pressures, with substantial reductions in midfoot and heel regions and frequent MMP and PP decreases exceeding 45 percent during walking.

**Conclusion:**

Integrating patient-specific geometry with active pneumatic offloading enhances plantar pressure redistribution and may help prevent DFUs.

## Introduction

Diabetes has become a global health crisis, affecting 589 million adults between the ages of 20 and 79 and creating an economic burden exceeding USD 1 trillion.^1^ According to the CDC, about 38.4 million Americans have diabetes, which corresponds to 11.6% of the entire U.S. population.^2^ One of the most serious complications is diabetic foot ulcers (DFUs), with approximately 1.6 million new cases annually and a lifetime risk of 19–34%.^3,4^ DFUs account for approximately 85% of diabetes-related amputations,^5^ and post-DFU mortality remains alarmingly high, with nearly 30% of patients dying within five years—survival outcomes comparable to those seen in cancer.^6^ These figures highlight the urgent need for effective offloading strategies to prevent diabetic foot ulcers (DFUs), as recommended by the International Working Group on the Diabetic Foot (IWGDF), in order to reduce morbidity, mortality, and healthcare costs.^7^

The concept of offloading in diabetic foot arose with the recognition that excessive and repetitive plantar pressure, along with shear forces, plays a major role in ulcer development and delayed healing.^8–10^ This understanding informs both therapeutic and preventive offloading strategies, which differ in their clinical purpose. Therapeutic offloading methods, such as knee-high devices like Total Contact Casting (TCC), were historically introduced for active ulcer treatment and remain the gold standard for managing neuropathic plantar DFUs.^11,12^ Removable peg insoles (“peg assist” insoles) similarly target localized pressure beneath an existing ulcer and are primarily used during wound healing.^7,13^ In contrast, preventive offloading approaches aim to reduce plantar pressure and shear forces before tissue breakdown occurs. Therapeutic footwear with insoles is designed for long-term preventive management of diabetic foot complications and is widely used in clinical practice due to its ease of implementation, improved comfort, and suitability for sustained preventive care.^14–16^

Custom-made insoles are generally more effective than standard insoles in reducing plantar pressure and preventing diabetic foot ulcers in high-risk patients, such as those with neuropathy or a previous ulcer history.^16–19^ Different combinations of materials, such as ethylene-vinyl acetate (EVA), polypropylene, and neoprene, along with various manufacturing techniques like molding, vacuum forming, and additive manufacturing, have been used to create insoles.^13,20,21^ They are generated based on 3D foot shape and plantar pressure maps, either using a single input or a combination of both.^22–24^ Clinical guidelines recommend custom-made insoles or footwear for patients with severe risk factors, while standard options may be acceptable for moderate-risk individuals when cost or access is a concern.^12^

Recently, insoles that can provide periodic active offloading have been studied and developed. Hemler et al.^25^ introduced an intelligent in-shoe insole system that uses magnetofluid materials and achieved peak pressure reduction through active pressure offloading features. Similarly, Erel et al. developed a pneumatically controlled air-cell-based insole comprising seven cells that span the entire plantar region of the foot and effectively reduce peak pressure in offloaded areas.^26^ Although recent studies indicate growing interest in pneumatically actuated insoles, many existing designs either fail to cover the entire plantar region or do not achieve substantial peak pressure reduction.^27,28^ One common aspect of all reported pneumatic insoles is their designs, which are based on generic foot geometries, similar to non-customized commercial insoles. To the best of the authors’ knowledge, no prior work has examined a custom-made insole with active offloading capability, and the present study addresses this gap by evaluating such an insole and assessing its effectiveness in reducing plantar pressure.

Building on evidence that custom insoles can redistribute plantar pressure effectively and that active offloading insoles can reduce peak pressure at targeted sites, this study examines the performance of a custom-made active insole compared with a non-custom active insole under static (standing) and dynamic (walking) conditions across key plantar regions. The manuscript begins with an overview of the pressure-alternating insole system, followed by descriptions of the experimental setup and the testing protocols used for both static and dynamic evaluations. Subsequent sections outline the data-processing procedures and statistical methods employed to quantify plantar-pressure outcomes. The results section then presents maximum average cell pressures and peak sensor pressures before and after offloading for both insole designs. Finally, the manuscript concludes with a discussion of the implications of these findings for the prevention of diabetic foot ulcers.

## Materials and methods

### Overview of pressure alternating shoe insole system

**Figure 1.**
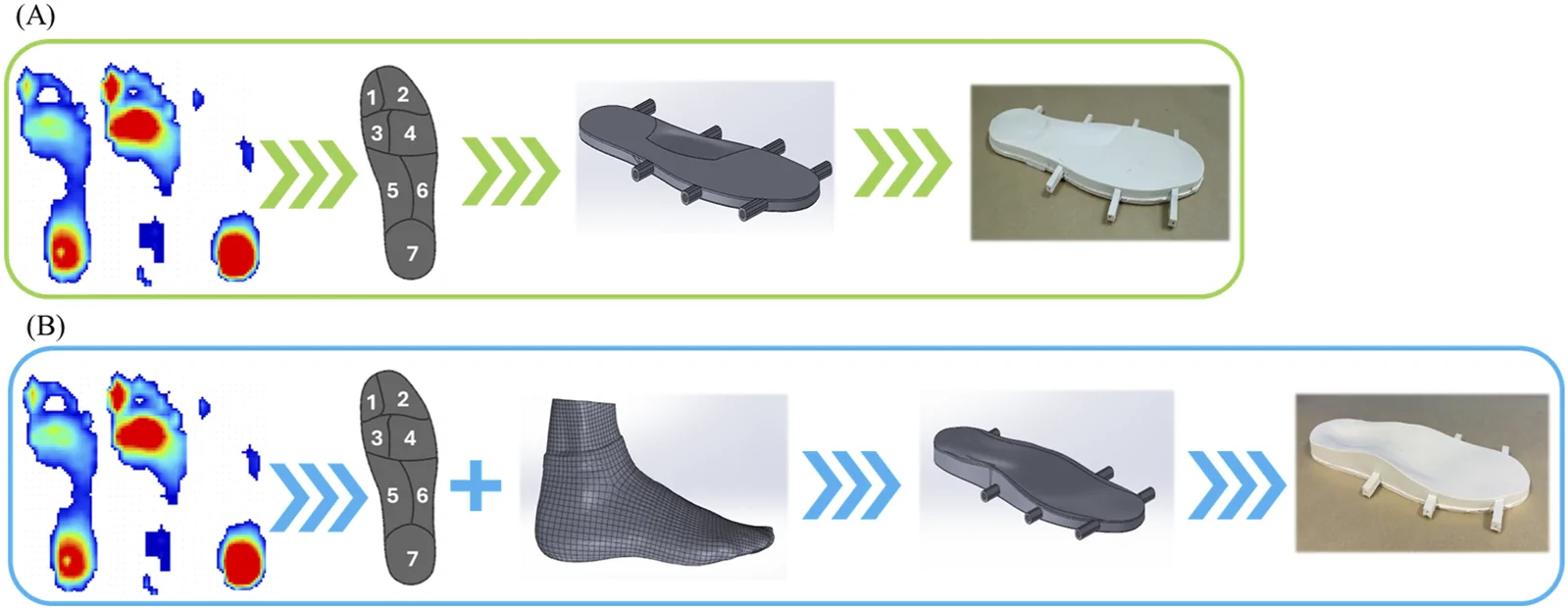
Fabrication workflow for (A) the non-custom flat insole and (B) the custom-contoured insole.

A comprehensive description of the insole system framework and its operational principles was published previously by Erel et al.^26^ In the present study, two types of pressure alternating insoles were evaluated: non-custom flat (Figure 1A) and custom-contoured (Figure 1B). The primary distinction between these designs lies in their design methodology. Both insole types incorporate seven air cells, which are generated based on plantar pressure distributions across gait phases (shown on the left side of Figure 1). These seven enclosed air cells are strategically located at plantar regions identified as critical sites for ulceration risk: Cells 1 and 2 in the toe regions, Cells 3 and 4 beneath the metatarsal heads, Cells 5 and 6 within the midfoot, and Cell 7 under the heel. Given that the main focus of this study is to evaluate the effect of custom insoles contoured to foot geometry, other design features, such as cell layout, were kept the same for both insoles.

The non-custom flat insole has a flat surface except in the arch region, while the custom insole features a fully contoured surface derived from the foot 3D-scan of the one individual who served as the single participant for the study. Thus, the distinction between the two designs lies not in cell placement but in the surface geometry used to construct the insole. Figure 1A illustrates the fabrication workflow for the non-custom flat insole, which is constructed using only interface pressure maps, while Figure 1B shows the workflow for the custom-contoured insole, which incorporates both the pressure map and the three-dimensional foot scan.

Each air cell is capable of holding pressurized air and is individually regulated through an integrated system comprising a pump, pressure sensors, and solenoid valves. This configuration enables selective inflation or deflation of each cell, while allowing continuous monitoring of internal pressure to ensure precise modulation of plantar loading [Insert Figure 1].

### Experimental design

Static and dynamic tests were performed on both insoles. A single participant (male, 61 kg, men’s shoe size 10, 65 years old, 5′5″) completed all testing sessions. Both non-custom flat and custom-contoured insole designs were assessed under two initial cell pressure settings of the insoles: 1.5 psi (10.34 kPa) and 2.0 psi (13.78 kPa). These pressure values were selected based on the simulation of the clinical scenarios conducted in a previous study.^26^ Cells 1, 3, 4, and 7, corresponding to regions at high risk for DFU formation, were selectively offloaded one at a time during testing.

**Figure 2.**
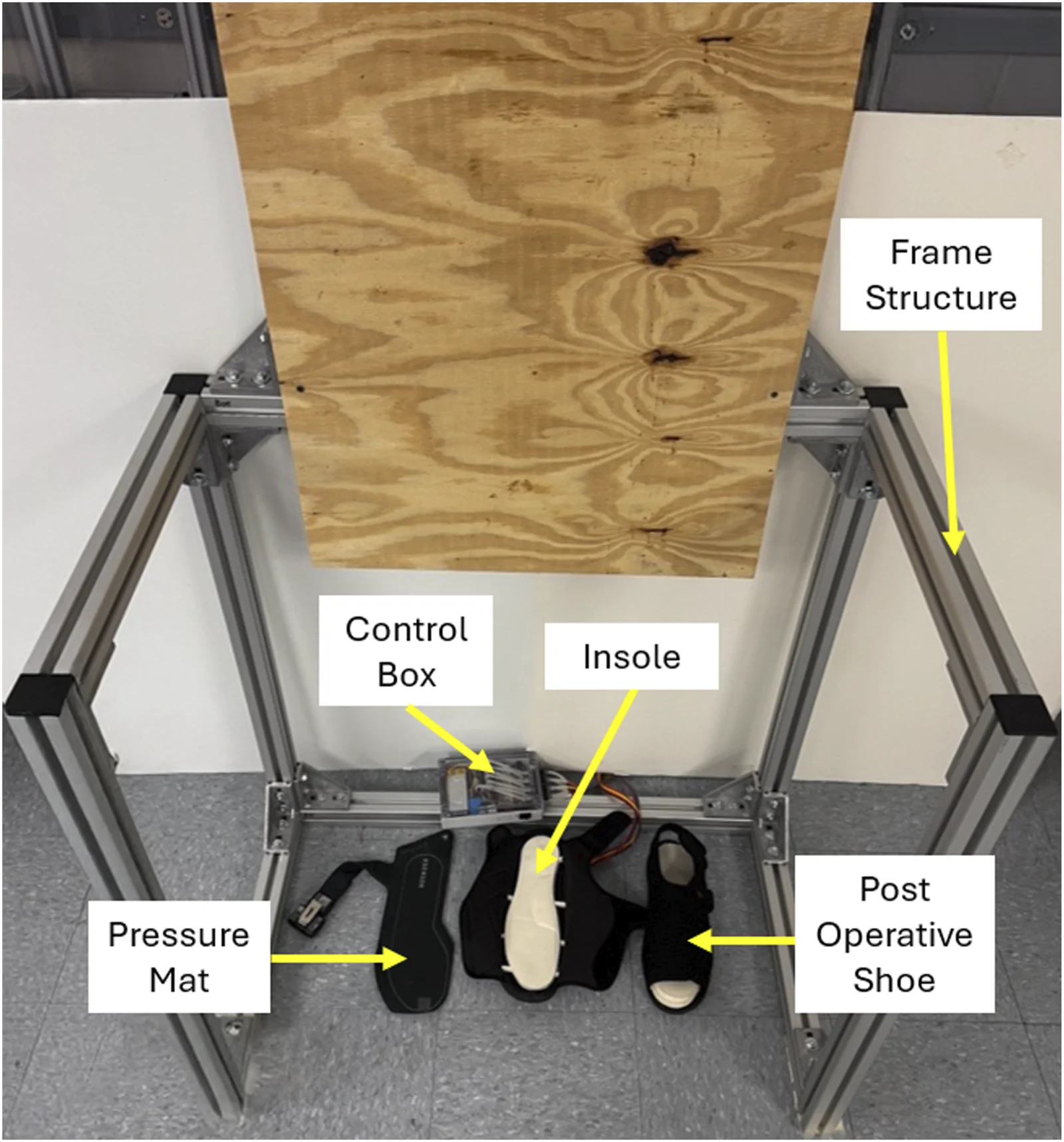
Static testing setup with the post-operative shoe, pressure mat, insole, and control box.

Before each trial, each insole was placed in a postoperative shoe and inflated to the predefined internal pressure. To maintain equal foot elevation and overall stability, insoles were positioned in both the right and left postoperative shoes and inflated to the same baseline pressure. Plantar interface pressure measurements were then collected on only the right foot using an XSensor® shoe size interface pressure sensor (spatial resolution: 7–9.4 mm; sampling rate: 100 Hz) positioned between the insole and the participant’s foot. The postoperative shoe, interface pressure sensor, and control box used to operate the insoles and execute the offloading function during the trials are shown in Figure 2.

### Testing protocols

Static (standing) and dynamic (walking) tests followed an identical offloading sequence consisting of six consecutive 30-second stages: (1) no cells offloaded, (2) Cell 1 offloaded, (3) Cell 3 offloaded, (4) Cell 4 offloaded, (5) Cell 7 offloaded, and (6) a final period with no cells offloaded. For each insole type, trials were conducted at initial cell pressures of 1.5 psi and 2.0 psi, resulting in four total trials across the two insole types and two pressure conditions. After the participant stepped onto the insole, interface pressure and internal cell pressures were continuously recorded throughout each trial. Beginning in the next section, the non-custom flat insoles are referred to as Flat 1.5 and Flat 2.0, and the custom contoured insoles as Custom 1.5 and Custom 2.0, where 1.5 and 2.0 denote the initial cell pressure values. This naming convention is used for clarity and brevity.

#### Static testing

Four static trials were performed using a frame structure constructed from aluminum profiles with a wooden backrest to ensure a consistent upright posture (Figure 2). Each static trial lasted 180 s, during which the participant remained standing while the offloading sequence was applied [Insert Figure 2].

#### Dynamic testing

Four dynamic trials were conducted on a treadmill. After stepping onto the insole, the participant walked on the treadmill at a speed setting of 0.536 meters/second, and the same six-stage offloading sequence was applied while the participant continued walking.

### Data and statistical analysis

**Figure 3.**
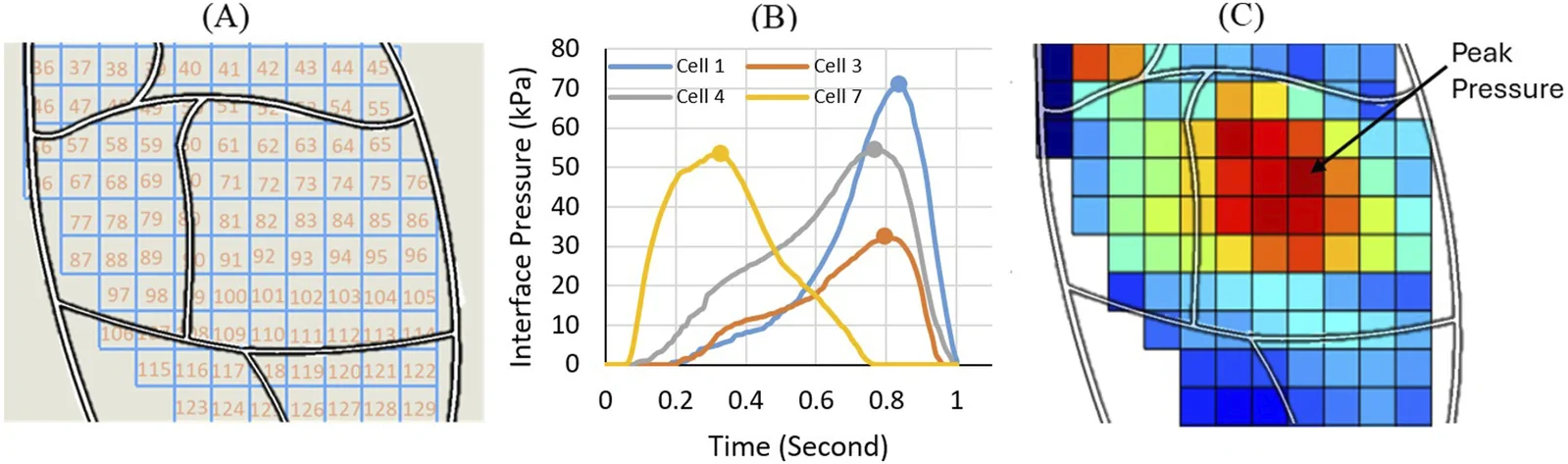
(A) Interface pressure mat sensor locations and corresponding insole-cell locations, (B) cell interface-pressure values during gait (walking) with maximum average-pressure points indicated, and (C) highest pressure reading from a single sensor.

XSensor shoe-size pressure mat contains 235 sensors, each corresponding to different locations of the foot (Figure 3A). Interface pressure recordings were processed in MATLAB to generate detailed pressure maps and to quantify cell-specific interface pressures. Because the primary objective of this study was to evaluate the effectiveness of cell offloading between the custom insole versus the non-custom insole, interface pressures in the regions corresponding to Cells 1, 3, 4, and 7 were extracted for analysis.

To ensure clarity in reporting, the manuscript employs defined terminology to characterize the influence of offloading interventions on plantar pressure profiles. The **Mean Pressure (MP)** was calculated by summing all sensor readings within a given cell and dividing by the number of sensors in that cell. Under dynamic conditions, MP were averaged across 12 gait cycles and plotted by normalizing the gait cycle time (0–1 s). The **Maximum Mean Pressure (MMP)** was defined as the highest MP value observed within each cell during the gait cycle and was computed using the same 12-step dataset for dynamic trials and a 20-second recording window for static trials (Figure 3B). The **Peak Pressure (PP)** represents the single highest sensor reading recorded within each cell region. For dynamic walking, PP was identified as the maximum sensor value within each gait cycle and then averaged across 12 steps; for static standing, PP was taken as the maximum sensor reading within the 20-second window (Figure 3C) [Insert Figure 3].

Statistical analyses were performed to assess changes in MMP and PP before and after offloading for each insole under both initial cell-pressure settings. Standard deviations, mean differences (MD), and corresponding *p*-values were computed to quantify variations in interface pressure. To determine whether offloading produced statistically significant differences relative to the before offloading condition, paired *t*-tests were conducted for both static and dynamic trials. Statistical significance was defined as a two-sided *p*-value < 0.05. All analyses were carried out using MATLAB R2022b.

## Results

### Static testing

Static tests were conducted with the participant standing to evaluate the offloading efficiency of two insole types at initial cell pressures of 1.5 and 2.0 psi. Table 1 presents the before- and after-intervention MMP interface pressure values for the offloaded cells (cells 1, 3, 4, and 7), along with the standard deviations, mean differences (MD), and p-values.

**Table 1.** MMP results (kPa) for flat and custom insoles at 1.5 psi and 2.0 psi during static testing.

​	Flat 1.5				Custom 1.5			
	*Before*	*After*	*MD*	*p-value*	*Before*	*After*	*MD*	*p-value*
Cell 1	10.38 ± 0.09	3.32 ± 0.00	7.06	0.059	8.78 ± 0.87	1.56 ± 0.04	7.21	0.057
Cell 3	14.50 ± 0.20	3.04 ± 0.00	11.46	<0.001	8.29 ± 0.22	1.78 ± 0.00	6.51	0.009
Cell 4	18.02 ± 0.31	1.04 ± 0.00	16.97	<0.001	12.54 ± 0.24	0	12.54	<0.001
Cell 7	27.83 ± 0.24	9.89 ± 0.10	17.9	<0.001	22.05 ± 0.60	4.69 ± 0.28	17.43	<0.001

​	Flat 2.0				Custom 2.0			
Cell 1	7.89 ± 0.07	1.22 ± 0.00	6.68	0.089	9.15 ± 0.21	1.74 ± 0.00	7.41	0.055
Cell 3	9.22 ± 0.24	1.45 ± 0.00	7.77	0.007	8.18 ± 0.39	0.74 ± 0.00	7.44	0.005
Cell 4	22.63 ± 0.38	0.49 ± 0.08	22.13	<0.001	16.79 ± 0.29	0	16.79	<0.001
Cell 7	31.28 ± 0.22	15.79 ± 0.18	15.47	<0.001	23.61 ± 0.20	12.29 ± 0.45	11.32	<0.001

**Figure 4.**
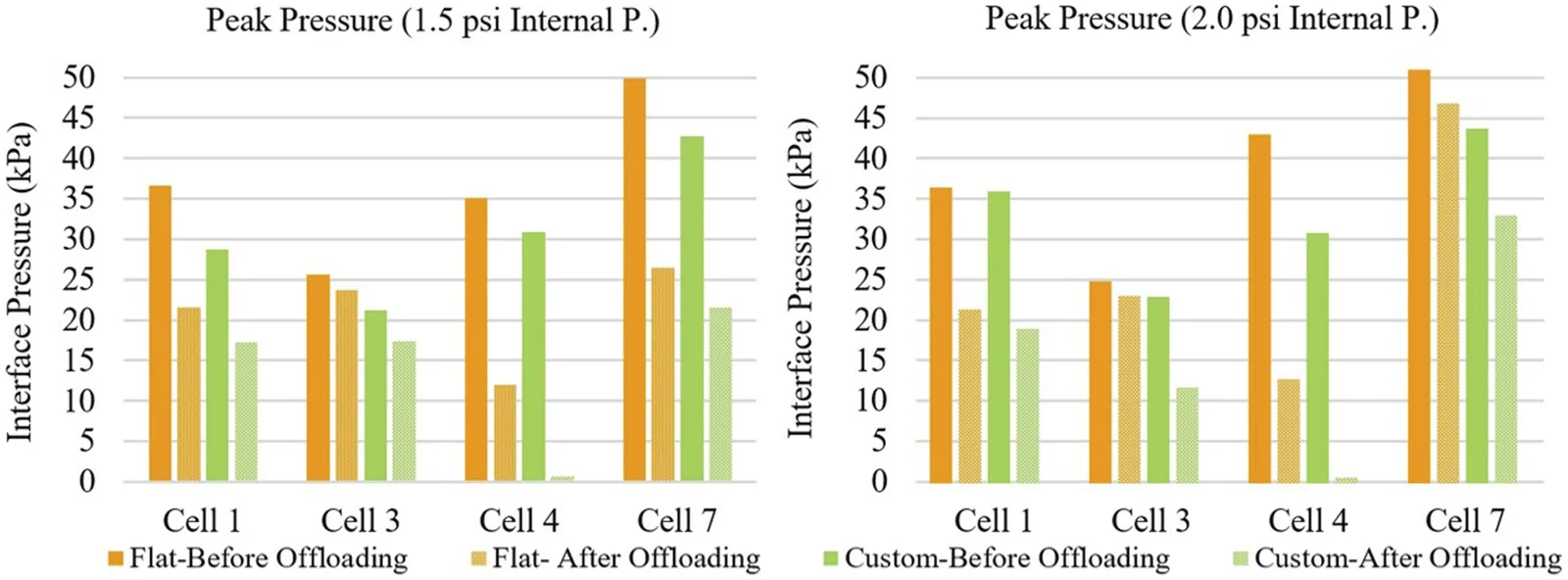
Peak pressures for flat and custom cells at 1.5 psi and 2.0 psi, shown before and after offloading during static testing.

For both the 1.5 psi and 2.0 psi conditions, the custom insole shows lower before and after offloading pressure values for the selected cells compared with the flat insole. The only exception occurred at Cell 1 at 2.0 psi, where the non-custom flat insole produced a lower value than the custom insole. Additionally, the custom insole at both initial pressures completely offloaded the Cell 4 regions, resulting in zero interface pressure after offloading. Beyond these patterns, Cell 7 consistently exhibited the highest pressures across all conditions, yet the after-offloading pressure values for the custom insole were lower than those observed for the flat insole. The small standard deviations relative to the large mean differences confirm that the observed reductions were not attributable to measurement variability. While Cell 1 exhibited the highest p-values (up to 0.089), likely due to less sensor contact and consequently lower sampling density, the p-values for all other cells were well below conventional significance thresholds, indicating that the pressure reductions observed in these regions were statistically significant [Insert Figure 4].

Figure 4 presents the peak pressure (PP) values for the flat and custom insoles at internal pressures of 1.5 psi and 2.0 psi. Across both pressure conditions, the custom insole consistently exhibits lower PP values than the flat configuration, both before and after the offloading conditions, excluding cell 1 at 2.0 psi. Similar to the MMP results, the PP in Cell 4 for the custom insole drops to zero after offloading, indicating complete unloading of this region at the sensor level and confirming that no plantar contact occurs between the Cell 4 region of the foot and the insole during offloading. When comparing the custom insole across the two internal pressures, the 1.5 psi condition produces lower PP values than the 2.0 psi condition before and after offloading. Overall, custom geometry lowers interface pressure more effectively in reducing localized PP during the standing condition. The maximum standard deviations observed were 0.22 kPa for flat 1.5, 0.94 kPa for flat 2.0, 0.86 kPa for custom 1.5, and 0.93 kPa for custom 2.0. Although these values can be observed as nearly a single line relative to the measured peak pressure values, error bars were not included in Figure 4 for clarity.

### Dynamic testing

We evaluated both non-custom flat insoles and custom-contoured insoles, each tested with and without offloading features, during a series of dynamic walking trials. The insoles were inflated to two pressure levels: 1.5 psi (10.34 kPa) and 2.0 psi (13.78 kPa), and each pressure condition was tested in a separate trial.

**Figure 5.**
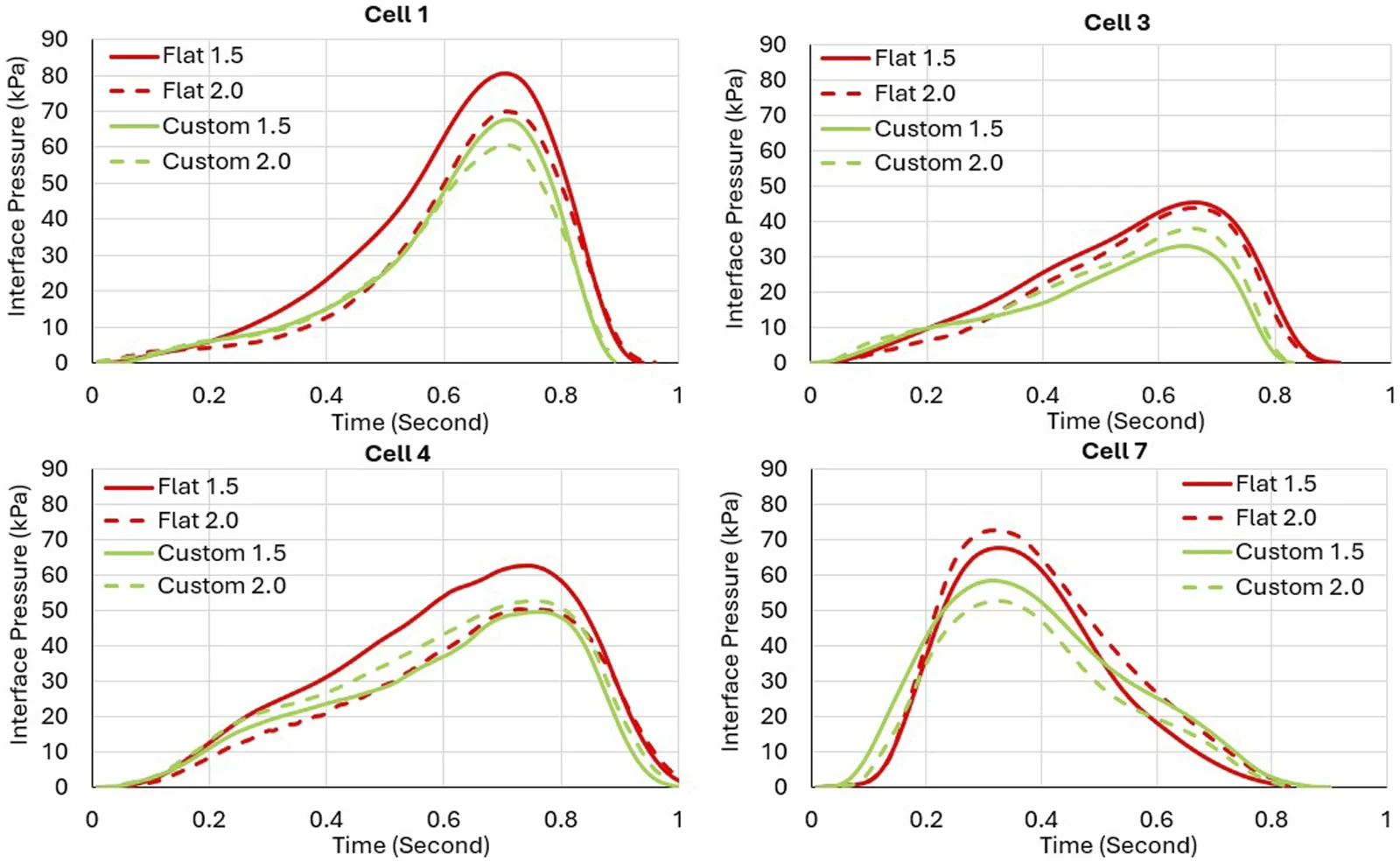
No Offloading - Interface pressure over the normalized gait cycle (0–1 s), averaged across 12 gait cycles, for Cells 1, 3, 4, and 7. Curves compare four insole configurations: Flat 1.5, Flat 2.0, Custom 1.5, and Custom 2.0.

Figure 5 presents the interface pressure–time curves across twelve consecutive walking steps under no-offloading conditions in the high-risk pressure ulcer regions (Cells 1, 3, 4, and 7). These data provide insight into interface pressure distribution across the insole designs. In most cells, the Flat 1.5 configuration (solid red line) produced the highest pressures, indicating less efficient load distribution. The only exception was Cell 7, where the Flat 2.0 condition (dashed red line) generated slightly higher peak pressures. In contrast, both Custom 1.5 and Custom 2.0 configurations (green lines) consistently exhibited lower pressures across the gait cycle, demonstrating that the contoured geometry improves load redistribution compared to the flat insole even during inactive offloading conditions. Within the custom conditions, Custom 2.0 showed the lower pressure profile for Cells 1 and 7, whereas Custom 1.5 produced lower pressures in Cells 3 and 4. Overall, these patterns suggest that flat insoles tend to transmit more force directly to the interface, while custom-contoured insoles help absorb and redistribute body weight more effectively during walking, when no offloading is applied [Insert Figure 5].

Table 2 shows that the Custom 1.5 and Custom 2.0 insoles reduce MMP more effectively than the flat insoles before offloading. After offloading, the custom 1.5 insole provides the lowest MMP in cells 1, 3, 4, and 7, whereas the flat insoles consistently produce higher pressures across regions. The largest overall reductions (mean difference) occurred with the custom 1.5 and flat 2.0 conditions, each showing average decreases of roughly 46–48%, with the other two conditions (custom 2.0 and flat 1.5) still producing meaningful but slightly smaller reductions. The absolute mean differences often exceeded 20 kPa and were consistently larger than the corresponding standard deviations, indicating that the reductions were substantial and well above expected measurement variability. Most comparisons reached statistical significance, and even the two cases with p = 0.07 showed clear downward trends that would likely reach significance with additional data.

**Table 2.** MMP results (kPa) for flat and custom insole for each cell during dynamic testing.

​	Flat 1.5				Custom 1.5			
	*Before Offld*	*After Offld*	*MD*	*p-value*	*Before Offld*	*After Offld*	*MD*	*p-value*
Cell 1	81.46 ± 4.44	64.93 ± 9.53	16.52	0.03	69.51 ± 4.64	38.12 ± 4.96	31.43	0.04
Cell 3	45.88 ± 2.92	24.80 ± 1.57	21.06	0.04	33.54 ± 1.14	17.61 ± 1.23	15.95	0.03
Cell 4	63.87 ± 2.94	37.47 +3.13	26.41	0.04	53.15 ± 2.65	26.78 ± 3.33	26.39	0.01
Cell 7	69.06 ± 4.52	55.16 + 3.97	13.90	0.03	58.27 ± 5.38	30.09 ± 5.64	28.16	0.01

​	Flat 2.0				Custom 2.0			
Cell 1	70.96 ± 8.06	47.53 ± 2.94	23.44	0.07	61.91 ± 4.84	45.54 ± 3.39	16.4	0.03
Cell 3	44.32 ± 2.80	20.99 ± 3.49	23.29	0.03	38.47 ± 2.44	24.91 ± 2.49	13.55	0.07
Cell 4	51.86 ± 2.95	27.44 ± 1.83	24.41	0.04	52.83 ± 1.44	30.97 ± 1.90	21.89	0.03
Cell 7	73.99 ± 4.15	34.20 ± 4.39	39.73	0.04	53.53 ± 2.00	32.55 ± 4.47	20.99	0.02

**Figure 6.**
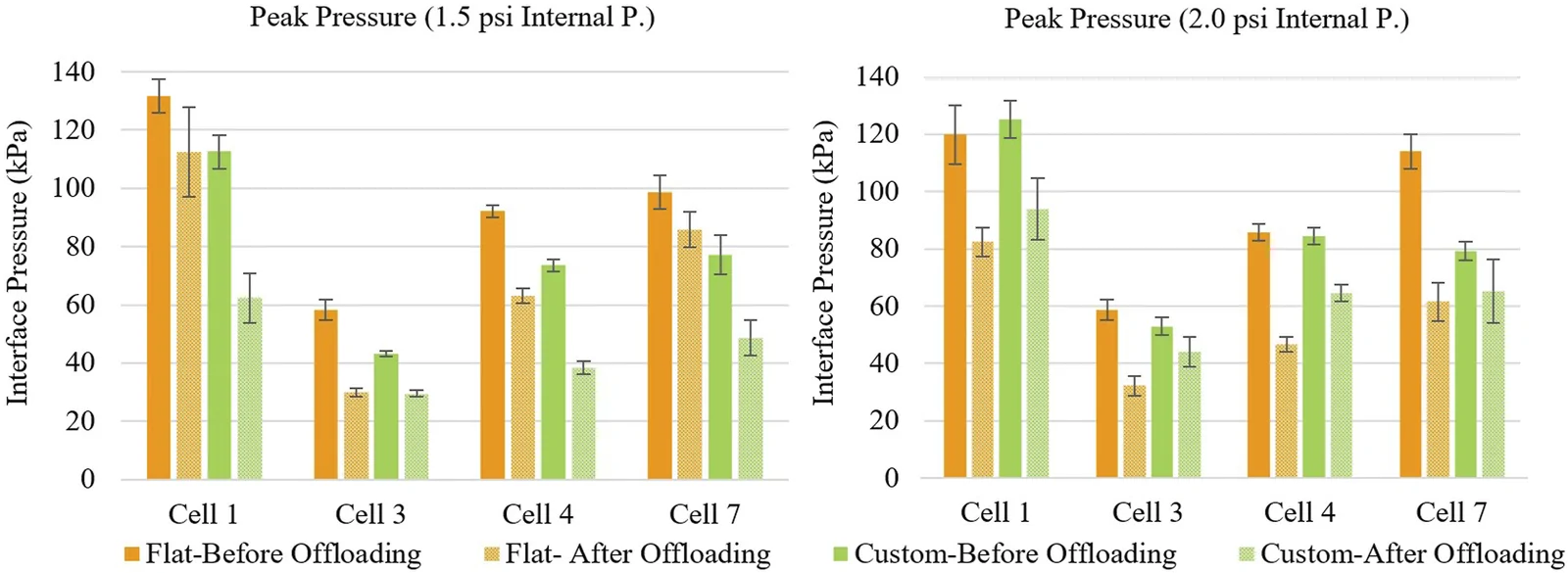
Peak interface pressures for flat and custom cells at 1.5 psi and 2.0 psi, shown before and after offloading during dynamic testing.

Figure 6 presents the peak pressure (PP) values before and after offloading for the dynamic cases. Across both internal pressure cases, the custom-before conditions generally exhibit lower PP values than the flat-before conditions, with the exception of Cell 1 at 2.0 psi. After offloading, the differences between configurations become clearer: the custom 1.5 psi condition consistently produces the lowest PP values across all evaluated cells, demonstrating the greatest pressure reduction. In contrast, the flat 1.5 psi insoles show smaller reductions and maintain higher PP values following offloading. At 2.0 psi, the differences between the flat and custom insoles are less pronounced, indicating more similar performance at this higher internal pressure. Overall, these results show that the custom-contoured insoles at 1.5 psi provide the most favorable pressure-redistribution performance. Notably, all PP values remain below the peak-pressure thresholds (∼200 kPa) reported in the literature as critical for preventing DFU formation.^29^ [Insert Figure 6]. Additionally, interface pressures on the midfoot and lesser toes corresponding to Cell 5, Cell 6, and Cell 2 were evaluated for both the flat and custom insoles throughout the dynamic testing protocol to ensure that offloading specific regions did not produce unintended pressure increases elsewhere. The maximum pressure recorded among flat and custom cases at Cell 2 was 32.51 kPa, at Cell 5 was 29.07 kPa, and at Cell 6 was 42.22 kPa, all of which remained below established peak-pressure threshold values. These findings confirm that offloading targeted cells does not result in harmful pressure elevations in adjacent, no offloaded regions.

## Discussion

During static testing, the custom insole design led to complete offloading at Cell 4, with both MMP and PP values dropping to zero at 1.5 psi and 2.0 psi. This response was not seen in the flat insole or in the other cell locations. The most likely explanation is that the neighboring custom cells were large and supportive enough to take on the load normally carried by Cell 4. In particular, Cell 2 and Cell 6, in front of and behind Cell 4 respectively, appear to have absorbed the redistributed pressure, allowing Cell 4 to remain unloaded. This suggests that the shaping and sizing of the custom cells were effective in directing force away from the targeted region. The same pattern observed in the PP measurements further supports the conclusion that the custom geometry succeeded in fully relieving pressure at Cell 4 without disrupting overall load distribution.

Across all four cells, the pressure–time curves (Figure 5) exhibit the typical stance-phase profile, but the flat insoles consistently generate higher peak pressures than the custom configurations. The normalized pressure-over-time plots further show that the custom-contoured insoles produce both lower peak values and smaller overall pressure integrals across the gait cycle, indicating more efficient redistribution of plantar load. Cells 1 and 3 show noticeably reduced mid-stance peaks with the custom geometry, suggesting improved attenuation of localized forces during early and mid-loading. In Cell 7, the custom insole curves (green solid and dashed lines) display a broader and more prolonged pressure profile compared with the flat insole, indicating that heel loading is sustained for a longer portion of the stance phase; this extended contact period helps distribute load more evenly and contributes to lowering mid-stance peak pressures.

When comparing the MMP and PP (Table 2 and Figure 6) trends at 1.5 and 2.0 psi during dynamic testing, increasing internal pressure improved the performance of the non-custom flat insole by reducing interface pressure values, consistent with earlier findings.^26^ In contrast, the custom-contoured insoles showed the opposite pattern, with higher internal pressure lessening their offloading benefit. Although optimizing internal pressure was not the focus of this study, the results suggest that adjusting pressure levels based on users’ weight and foot size could enhance offloading effectiveness using the current pressure-alternating insole system without additional hardware. Moreover, because the design enables independent control of each air cell, exploring differential pressurization strategies may further improve overall offloading performance.

A comparison of peak plantar pressure (PP) with existing literature (Hemler et al.^25^ and Shuang et al.^27^) suggests that our air cell–based insoles produce substantially lower interface pressures, with PP values around 130 kPa compared to approximately 270 kPa reported in previous studies. However, a portable insole system developed by Castro-Martins et al., which incorporates four air-cell, reported similar average and peak pressure values on heel, midfoot, metatarsal, and toes regions,^28^ supporting the plausibility of our findings. These results indicate that air cell–based insoles have the potential to maintain plantar pressures below ulcer-risk thresholds while providing adaptable and targeted offloading. However, the present findings are limited by evaluation in a single, low-body-weight participant. Given that plantar pressure is strongly influenced by body mass, foot geometry, and shoe size, future studies involving larger and more diverse cohorts are required to determine whether these advantages persist across broader populations.

The plantar pressure values observed in this study were lower than some reported in clinical and research settings, and a verification of the data processing procedures confirmed that no methodological or computational issues contributed to these findings. Instead, several biomechanical and experimental factors plausibly explain the reduced magnitudes. The participant’s relatively low body mass (61 kg) combined with a large shoe size (US men’s size 10) increased plantar contact area, thereby lowering average and peak interface pressures. In addition, the compliant air cell insole material distributed load more evenly across the plantar surface, consistent with previous findings^26^ using flat air-cell insole. The participant also walked at his comfortable pace, which was slower than typical walking speeds reported in the literature and is known to reduce plantar loading through lower ground-reaction forces and longer stance times.^30^ Although slow walking alone does not fully account for the magnitude of reduction, its influence combined with the participant’s anthropometrics and the compliant insole material, provides a plausible explanation for the lower plantar pressure values observed in this study.

Preventive offloading strategies for individuals at risk of diabetic foot ulceration typically focus on reducing plantar pressure and shear through therapeutic footwear, custom insoles, and targeted design features that redistribute load across the foot. Existing literature demonstrates that such interventions can lower peak plantar pressures and reduce ulcer recurrence, yet their effectiveness depends heavily on consistent use and the ability to achieve adequate pressure reduction across multiple high-risk regions.^14–16^ The proposed intervention aligns with these established preventive approaches by aiming to redistribute plantar loads before tissue breakdown occurs, while offering an added benefit through its ability to more effectively target pressure redistribution in specific anatomical regions identified as vulnerable due to the personalization capability. In addition, the device provides active and continuous offloading throughout gait, enabling real-time modulation of plantar loading rather than relying solely on static design features. This dynamic capability allows the intervention to adapt to changing loading patterns during the gait cycle, offering a more responsive approach than conventional preventive devices. By integrating targeted, personalized, active, and continuous offloading within a preventive framework, the intervention may complement current solutions and strengthen overall pressure-management strategies for individuals at elevated risk of ulceration.

## Conclusion

In this study, the custom-contoured insole was compared with a non-custom flat insole at internal cell pressures of 1.5 psi and 2.0 psi under both static and dynamic testing conditions. Under static loading, the custom insole consistently demonstrated better pressure-relief performance compared to the flat insole at both pressure levels. During dynamic testing, the Custom 1.5 psi insole again showed reliably improved outcomes, whereas the custom and flat insoles exhibited mixed performance at 2.0 psi. Collectively, these findings highlight the enhanced offloading capability of the custom-contoured insole and are worth exploring further.

Air cell–based insoles also offer inherent advantages, including the ability to modulate pressure distribution dynamically, accommodate variations in gait and foot morphology, and deliver adaptable, targeted offloading to high-risk regions. Notably, even before offloading was activated (no offloading), low peak pressure values were observed, further supporting the potential of these designs. Together, these features make air-cell systems especially promising for reducing localized plantar loading and supporting long-term DFU prevention.

Overall, the results of this study demonstrate that actively offloading custom insoles were more effective than non-custom insoles in reducing both maximum mean pressure and peak pressure. Because both types of pressure contribute to the development of diabetic foot ulcers, a custom insole that more substantially reduces these pressures has the potential to lower the incidence of DFU. However, additional studies are needed to better understand how the pneumatic insole and its offloading capabilities affect other critical parameters, such as balance and blood perfusion. A prospective, adequately powered study is also warranted to validate the design and manufacturing framework, as well as the effectiveness of the custom-contoured active insole.
